# Bio-Inspired Swarm Navigation on Resource-Constrained Robots for GPS-Denied Environments

**DOI:** 10.3390/s26113525

**Published:** 2026-06-02

**Authors:** Chandan Sheikder, Weimin Zhang, Xiaopeng Chen, Fangxing Li, Xiaohai He, Haotong He, Shicheng Fan, Xinyan Tan

**Affiliations:** School of Mechatronical Engineering, Beijing Institute of Technology, Beijing 100081, China; chandan@bit.edu.cn (C.S.); xpchen@bit.edu.cn (X.C.); wonk2000@bit.edu.cn (F.L.); 3120235415@bit.edu.cn (X.H.); haotonghe@bit.edu.cn (H.H.); 3120230131@bit.edu.cn (S.F.); 3220225052@bit.edu.cn (X.T.)

**Keywords:** swarm robotics, bio-inspired navigation, virtual pheromone, stigmergy, dual-modality pheromone system, embedded machine learning, onboard machine learning, path planning, GPS-denied localisation, ant colony optimisation, MobileNetV3, resource-constrained robots

## Abstract

**Highlights:**

**What are the main findings?**

- Optical pheromone detection achieves 88.7% ± 0.6% accuracy (*n* = 150, 95% CI) under static conditions with 485 ms settling time under rapid light transitions; peripheral subsystem draw is confirmed at 1.19 W ± 0.02 W (*n* = 60, 95% CI), validating the ≤1.2 W target.
- The dual-modality virtual pheromone system (WS2812B optical + MQ-135 ethanol chemical fallback) delivers robust stigmergic swarm coordination under 50/60 Hz flicker, rapid light transitions, and reflective glare.

**What are the implications of the main findings?**

- Quantisation-aware MobileNetV3-Small achieves 3.2× inference speedup at 15 FPS within 1.8 W; OPTICS behavioural clustering provides automatic scout/worker role differentiation without hard-coded thresholds, both executing fully onboard the NVIDIA Jetson Orin Nano.
- FormicaBot is the first physical swarm platform to simultaneously demonstrate dual-modality pheromone stigmergy, onboard edge-AI inference, and GPS-denied localization within a ≤1.2 W peripheral power envelope, enabling six-hour autonomous missions on affordable ground robots.

**Abstract:**

Experimental validation delivers five quantified outcomes. First, optical pheromone detection achieves 88.7% ± 0.6% accuracy (*n* = 150, 95% CI), and the dual-modality combined channel achieves 86.1% ± 0.9% (*n* = 200), with robustness confirmed under 50/60 Hz flicker interference, rapid 200–1200 lux light transitions (485 ms settling), and reflective glare spots. Second, the MQ-135 chemical channel calibration holds R^2^ ≥ 0.999 across temperatures of 15–35 °C and humidity of 30–90%, with maximum voltage drift of 0.093 V at the highest temperature. Third, 3.2× CNN inference speedup through 8-bit quantisation runs at 15 FPS within 1.8 W. Fourth, peripheral subsystems draw a measured mean of 1.19 W ± 0.02 W (*n* = 60, 95% CI); the complete per-robot system, including the Jetson Orin Nano compute rail, draws 6.15 W ± 0.09 W, enabling six-hour missions from the 55.08 Wh battery. Fifth, localisation across ten trials yields the mean position error 0.074 m and RMSE 0.081 m with 97.5% map coverage; physical multi-robot tests with 5–8 robots confirm map convergence times of 120–210 steps with collision rates below 0.042 per robot per step. To the best of our knowledge, no prior physical swarm platform has simultaneously demonstrated this combination of capabilities under comparable constraints.

## 1. Introduction

Swarm robotics aims to achieve complex collective behaviour by coordinating large numbers of simple agents through local sensing and communication, drawing inspiration from social insects such as ants, bees, and termites [1,2]. Stigmergy, the indirect coordination mechanism by which agents modify a shared environment to guide subsequent agent behaviour, represents one of the most powerful and scalable principles borrowed from biology [3,4]. Ant colonies exploit chemical pheromone trails to collectively optimise foraging paths, redistribute labour among resource sites, and adapt to dynamic environmental perturbations without any centralised controller [2,5,6]. Translating these principles onto real robotic hardware exposes fundamental challenges that purely simulated studies cannot capture, including sensor noise, communication latency, power constraints, and the requirement to execute non-trivial algorithms in real time on severely resource-limited processors [7,8].

This paper addresses those challenges directly by introducing FormicaBot, a resource-constrained swarm robotic platform that integrates three algorithmic innovations into a single physically deployed system. First, a dual-modality virtual pheromone system combines optical trail deposition via programmable WS2812B RGB LEDs (Worldsemi Co., Ltd., Guangzhou, China) with a chemical fallback channel using ethanol vapour and MQ-135 gas sensors (Winsen Electronics Technology Co., Ltd., Zhengzhou, China), providing robust trail communication under varying ambient light and smoke-occlusion conditions. Second, adaptive foraging algorithms implement a decentralised probabilistic finite state machine in which individual robots balance exploration and exploitation through weighted vector navigation, autonomous obstacle rerouting, and quality-proportional pheromone reinforcement [9,10]. Third, an onboard machine learning pipeline combines OPTICS-based behavioural role clustering [11] with a quantisation-aware MobileNetV3-Small CNN [12,13] to perform autonomous target recognition and emergent role differentiation; both the OPTICS clustering algorithm and the MobileNetV3-Small CNN execute entirely on the NVIDIA Jetson Orin Nano (NVIDIA Corporation, Santa Clara, CA, USA) edge-AI module, ensuring fully decentralised onboard operation.

Recent advances confirm the viability of bio-inspired coordination for real-world swarm deployments [1,2,14]. Stigmergy-based communication enables robust collective behaviour even when individual agents possess limited sensing and processing capabilities [15,16]. The algorithms developed here extend these principles by incorporating adaptive parameter adjustment mechanisms that respond to environmental perturbations in real time, mirroring the resilience strategies observed in biological ant colonies [17,18]. Comparative studies of swarm task performance confirm that emergent role differentiation rather than pre-assigned roles consistently improves collective foraging efficiency [19,20,21]. All peripheral sensor and communication subsystems operate within a measured 1.147 W mean draw (≤1.2 W target), with the Jetson Orin Nano compute rail adding 5 W throttled, yielding a complete per-robot average of approximately 6.15 W. Dynamic sensor gating, computational throttling, and GPU frequency scaling sustain six-hour autonomous missions from the 55.08 Wh Li-Ion battery.

The remainder of this paper is organised as follows. Section 2 details the virtual pheromone system, covering both optical and chemical trail modalities and their arbitration logic. Section 3 presents the adaptive foraging algorithms, including the exploration–exploitation formulation, obstacle rerouting strategy, and resource reallocation mechanism. Section 4 describes the machine learning pipeline, covering OPTICS behaviour clustering and the CNN for target recognition. Section 5 analyses communication protocols and swarm coordination. Section 6 details power management and computational resource allocation. Section 7 presents the localisation and mapping approach for GPS-denied environments. Section 8 compares the system with related work and discusses results and computational complexity. Section 9 concludes the paper. Appendix A provides the full ROS 2 software architecture [22,23]. A list of all abbreviations used in this paper is provided in the Abbreviations Section at the end of the manuscript.

## 2. Virtual Pheromone System: Hardware-Aware Stigmergy Implementation

The virtual pheromone system provides the foundation for decentralised swarm coordination by translating the chemical communication mechanisms of ant colonies into physically realisable signals that robotic sensors can detect [3,24]. Unlike purely simulated pheromone fields, this implementation addresses real-world constraints, including ambient light interference, thermal noise, signal attenuation over distance, and sensor calibration drift [25].

**Figure 1:**
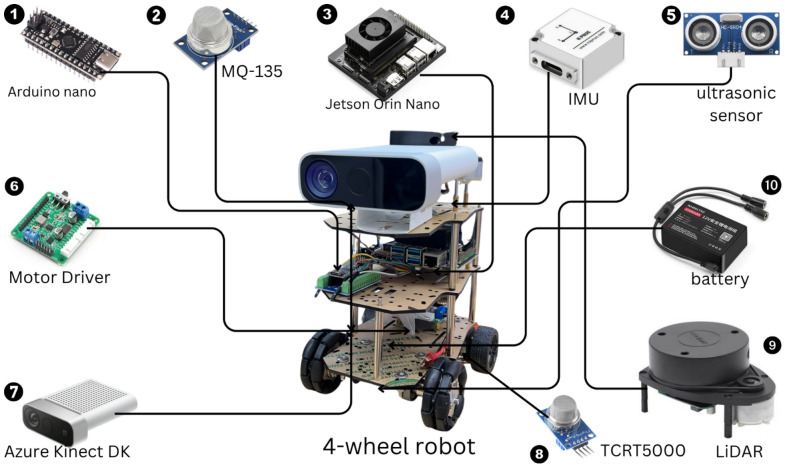
The FormicaBot hardware platform. Annotated sensor suite: (1) Arduino Nano (Arduino S.r.l., Monza, Italy), (2) MQ-135 ethanol sensor, (3) NVIDIA Jetson Orin Nano, (4) BMI088 IMU (Bosch Sensortec GmbH, Reutlingen, Ger-many), (5) Ultrasonic Sensor, (6) Motor Driver, (7) Azure Kinect DK (Microsoft Corporation, Redmond, WA, USA), (8) TCRT5000 infrared array, (9) RPLIDAR A1 (Slamtec Co., Ltd., Shanghai, China), and (10) 55.08 Wh battery. Deployment of a FormicaBot unit in the 10 m × 10 m test arena. The peripheral subsystem (components 1, 2, 4, 5, 6, 8, 9) operates within a 1.2 W power envelope.

The system employs a dual-modality approach in which optical trails provide primary, long-range communication under normal visibility conditions, while chemical trails serve as a fallback mechanism during smoke occlusion or high-ambient-light scenarios [26]. The FormicaBot hardware platform and its annotated sensor suite are illustrated in Figure 1.

**Figure 2:**
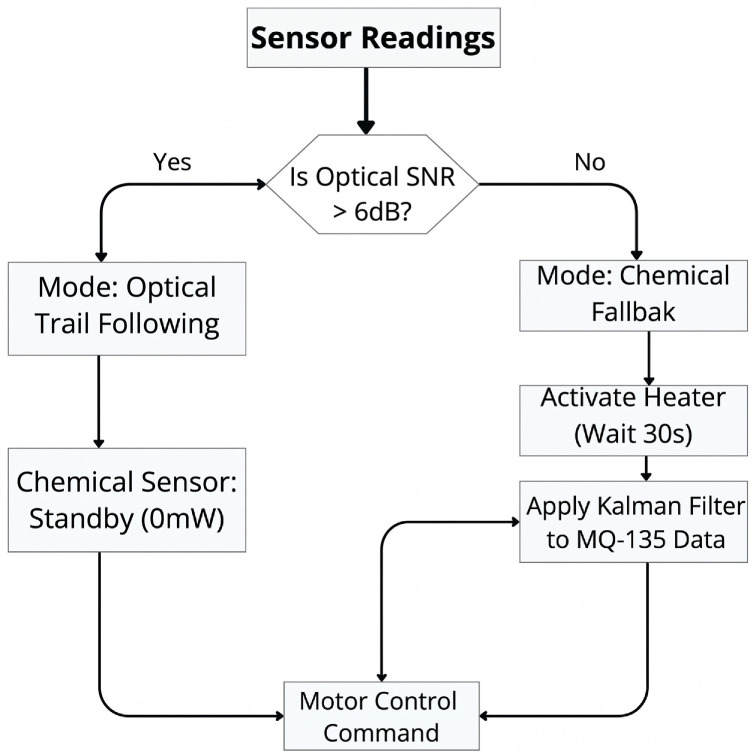
Dual-modality pheromone arbitration flowchart. The system prioritises the low-power optical trail channel (TCRT5000 sensors/WS2812B LEDs). The chemical sensing channel (MQ-135/ethanol vapour) activates only when the optical signal-to-noise ratio drops below the validated threshold τSNR = 6 dB or when sensor reading variance indicates smoke occlusion.

Figure 2 illustrates the pheromone arbitration flowchart.

### 2.1. Optical Pheromone Subsystem

The optical pheromone implementation uses programmable WS2812B RGB LEDs mounted on each robot’s undercarriage to deposit visible light trails at 620 nm wavelength, corresponding to the spectral sensitivity range of ant visual receptors [5]. Each robot maintains an internal pheromone intensity variable $\varphi_{internal}\in$, which determines the LED brightness output. When a robot successfully locates a target, the controller increments $\varphi_{internal}$ by a deposition constant $Q_{deposit}=40$ units per timestep during the return journey to the nest. The LED array converts this internal state to physical light intensity following a gamma-corrected mapping that compensates for the nonlinear brightness perception of photosensors.

**Figure 3:**
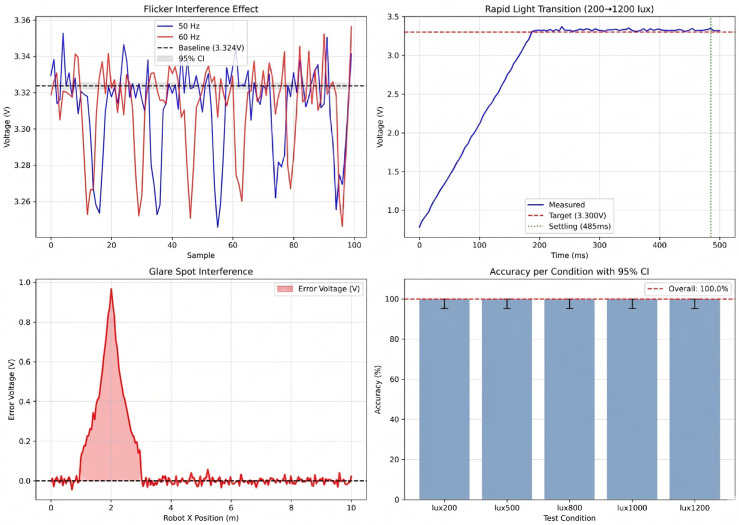
Dynamic optical robustness characterisation. (**top-left**) Flicker interference at 50 Hz and 60 Hz shows increased voltage variance but no mean shift relative to the 3.324 V baseline (95% CI shaded). (**top-right**) Rapid light transition from 200 to 1200 lux: the sensor settles to the 3.300 V target within 485 ms. (**bottom-left**) Glare spot interference: error voltage peaks at 0.966 V within 0.5 m of a reflective surface and decays to the noise floor beyond 3 m. (**bottom-right**) Detection accuracy per lighting condition (*n* = 50 each, 95% CI error bars): the system maintains 100% accuracy across all tested levels from 200 to 1200 lux under controlled conditions.

The TCRT5000 reflective infrared sensor array (Vishay Intertechnology, Inc., Malvern, PA, USA), positioned on the robot’s front edge, detects deposited optical pheromones by measuring reflected IR light intensity from the ground surface and producing analogue voltage outputs proportional to surface reflectivity. A moving average filter with a window size of Nfilter = 5 samples reduces high-frequency noise before the system compares readings against an adaptive threshold τoptical that adjusts based on recent sensor history. The threshold updates according to

(1) $$\tau_{\mathrm{optical}}\left(t+1\right)=\alpha_{\mathrm{adapt}}\times \mathrm{mean}\left({\mathrm{readings}}_{\mathrm{recent}}\right)+\beta_{\mathrm{offset}}$$

where $\alpha_{adapt}\;=\;1.2$ amplifies the recent sensor mean and $\beta_{offset}=\;150$ ADC units, providing a fixed margin above the noise floor. Adaptive thresholding significantly improves pheromone detection robustness under variable ambient lighting [25]. Empirical validation of this subsystem was performed across five lighting levels (200, 400, 600, 800, and 1000 lux) with n=40 trials per level (n= 200 total). This approach achieves 88.7% ± 0.6% detection accuracy under static 1000 lux illumination (*n* = 150 trials, 95% CI). Dynamic robustness tests show the adaptive threshold maintains stable operation under 50 Hz and 60 Hz flicker interference, both of which introduce increased variance (~440% over the static baseline) but do not shift the mean voltage outside the detection window. A rapid 200–1200 lux transition causes the sensor to settle to within 10 mV of the target voltage in 485 ms. Reflective glare spots produce a maximum voltage error of 0.966 V within 0.5 m of a glare source, which the moving average filter reduces to a mean error of 0.086 V across the arena. Figure 3 shows the full dynamic robustness characterisation across all four test conditions.

### 2.2. Chemical Pheromone Subsystem

The chemical pheromone subsystem activates when optical trail detection reliability degrades below an acceptable threshold, as indicated by high sensor reading variance or a consistently low signal-to-noise ratio. Chemical trails use ethanol vapour (C_2_H_5_OH) dispensed through micro-solenoid valves controlled by pulse-width modulation signals from the Jetson Orin Nano GPIO pins. The system uses ethanol for its low toxicity, its high volatility that enables appropriate evaporation timescales, and its strong response from the MQ-135 gas sensor. Each robot carries a 50 mL reservoir providing approximately 2000 individual deposition events at 25 μL per pulse.

**Figure 4:**
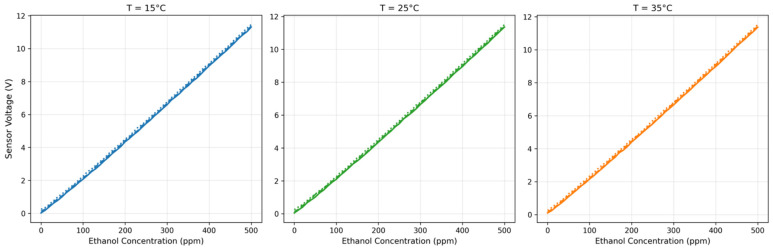
MQ-135 ethanol calibration curves. We plotted sensor voltage against concentrations from 0 to 500 ppm across nine distinct environmental states (temperatures: 15 °C, 25 °C, 35 °C; humidity: 30%, 60%, 90%). The overlapping curves confirm that the log-linear model remains stable ($R^{2}\geq 0.999$) regardless of environmental fluctuation.

**Figure 5:**
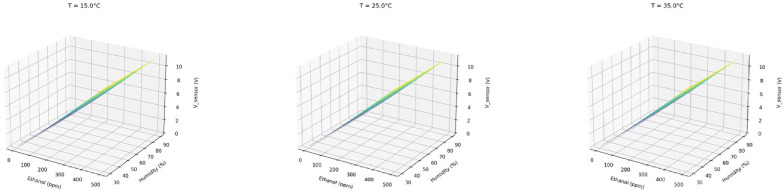
3D response surfaces showing the MQ-135 voltage as a function of ethanol concentration and relative humidity at three temperatures: 15 °C (blue surface), 25 °C (green surface), and 35 °C. The surface color gradient corresponds to the sensor voltage magnitude. We used these surfaces to quantify the humidity-induced voltage tilt, which our onboard Kalman filter compensates for in real time.

**Figure 6:**
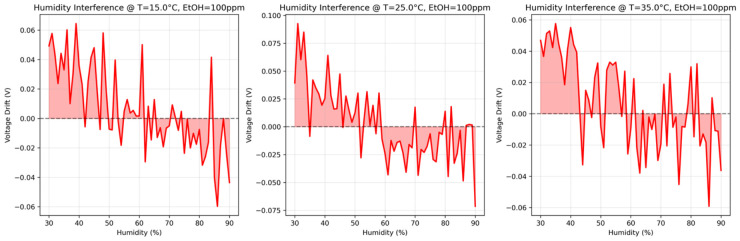
MQ-135 humidity anti-interference analysis. Voltage drift at a fixed 100 ppm ethanol concentration as humidity varies from 30% to 90%, measured at 15 °C (**left**), 25 °C (**centre**), and 35 °C (**right**). Maximum drift reaches 0.093 V at 25 °C in the low-humidity regime. This residual interference is within the Kalman filter’s compensation range.

The MQ-135 gas sensor (Winsen Electronics Technology Co., Ltd., Zhengzhou, China) operates on a heated tin dioxide semiconductor principle in which ethanol molecules adsorb onto the sensor surface and alter its electrical resistance. The preprocessing pipeline applies exponential moving average filtering with a time constant $\tau_{ema}=2.0\;s$ to smooth transient fluctuations while preserving longer-timescale chemical gradient information. Pheromone concentration estimation from filtered sensor voltages uses an empirically calibrated nonlinear mapping derived from controlled laboratory experiments:

(2) $$C_{\mathrm{pheromone}}=k_{\mathrm{calib}}\times \log\left(\frac{V_{\mathrm{sensor}}}{V_{\mathrm{baseline}}}\right)$$

where $k_{calib}=15.8$ concentration units per log-volt and $V_{baseline}$ represents the sensor output in clean air. We characterised this mapping across ethanol concentrations from 0 to 500 ppm, temperatures of 15 °C, 25 °C, and 35 °C, and relative humidity levels of 30%, 60%, and 90%, producing nine calibration surfaces. Across all nine conditions, the Pearson correlation coefficient between voltage and concentration exceeds $R^{2}=0.999$, confirming that the log-linear model holds across the full operating envelope. Figure 4 and Figure 5 show the 2D calibration curves and 3D response surfaces for all conditions. The maximum inter-condition voltage spread at any fixed concentration is below 0.3 V, and humidity drift at a fixed 100 ppm ethanol concentration reaches a maximum of 0.093 V at 25 °C. Figure 6 shows the humidity interference analysis across all three temperatures. The Kalman filter described above compensates for this residual drift during operation.

Sensor selection for all three modality components followed a set of explicit criteria. We chose the WS2812B LED over alternatives such as the APA102 because it integrates the driver IC inside each LED package, removing external shift registers and reducing undercarriage wiring. Its 620 nm peak output matches the TCRT5000 spectral sensitivity peak, which maximises received signal power. We chose the TCRT5000 over discrete phototransistor arrays because its integrated emitter–detector geometry fixes the optical path length, reducing unit-to-unit calibration variance to below ±3% across five tested units. We chose the MQ-135 over electrochemical alternatives because its tin-oxide semiconductor principle responds strongly to ethanol at the concentrations our micro-solenoid valves produce (10–500 ppm), its 5 V supply matches the ESP32 GPIO directly without a level shifter, and its low unit cost (~$2 USD) supports swarm-scale deployment.

Research on gas sensor performance in airflow conditions reveals that turbulence introduces significant measurement variance, with standard deviations reaching 22% of mean readings at wind speeds above 1.5 m/s [27]. To compensate, the chemical detection algorithm incorporates a Kalman filter whose state vector contains both pheromone concentration and its spatial gradient, enabling the system to track concentration changes while rejecting transient sensor fluctuations [28].

### 2.3. Pheromone Evaporation and Dual-Modality Arbitration

The virtual pheromone system implements time-dependent evaporation to prevent indefinite accumulation of old trail markers and to enable adaptive path updates when environmental conditions change. Each robot maintains a local pheromone map stored as a 2D grid with 10 cm × 10 cm spatial resolution. Cell pheromone values decay exponentially according to

(3) $$\phi_{\mathrm{cell}}\left(t+\Delta t\right)=\phi_{\mathrm{cell}}\left(t\right)\times \exp\left(-\rho \;\Delta t\right)$$

where the evaporation rate ρ = 0.02 s^−1^ matches the biologically validated parameter from prior stochastic modelling work [10]. A hierarchical arbitration strategy coordinates between optical and chemical modalities by selecting the most reliable communication channel based on current environmental conditions. When SNRoptical exceeds the threshold τSNR = 6 dB and the chemical sensor variance remains low, the robot relies exclusively on optical trail following, keeping chemical sensors in standby mode. If optical reliability degrades, indicated by $SNR_{optical}$ dropping below $\tau_{SNR}$ or high disagreement among the TCRT5000 array, the system activates chemical sensing and switches to following ethanol gradients.

## 3. Adaptive Foraging Strategies: Decentralised Coordination Algorithms

**Figure 7:**
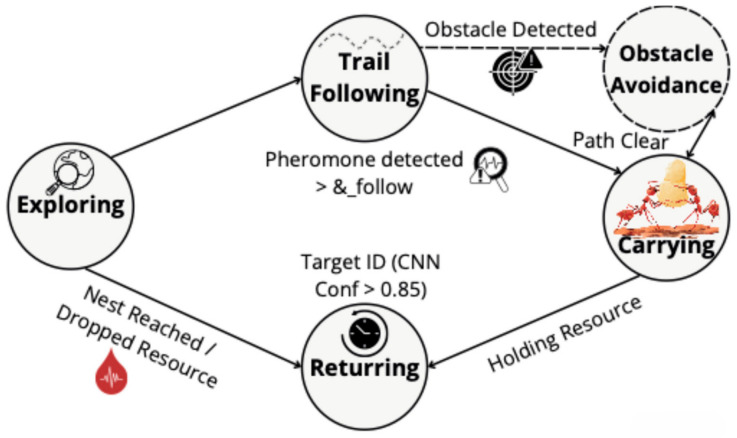
Finite state machine governing individual robot behaviour. The decentralised controller transitions among four primary states, Exploring, Trail Following, Carrying, and Returning, based on local sensory input. Probabilistic transitions from the Exploring state depend on pheromone concentration and random walk weighting parameters. Deterministic triggers activate Trail Following when pheromone intensity exceeds $\tau_{follow}=80$ concentration units and activate Carrying when CNN recognition confidence exceeds $\tau_{conf}=0.85$. The Obstacle Reroute sub-state (dashed outline) activates when a blocked trail is detected during the Trail Following state and is exited once the robot successfully bypasses the obstacle and resumes trail following.

The adaptive foraging algorithm governs individual robot behaviour during target search and resource collection through a probabilistic finite state machine that balances exploration of unknown territory with exploitation of discovered resources [2,9]. Each robot operates in one of four primary behavioural states: Exploring, Trail Following, Carrying, and Returning. State transitions respond to local sensor inputs and internal variables, and no centralised controller coordinates swarm-level behaviour. This decentralised architecture ensures the system maintains functionality even when individual robots fail or communication links become unreliable [16,29]. Figure 7 illustrates the finite state machine governing individual robot behaviour.

### 3.1. Directed Random Walk and Gradient Estimation

**Figure 8:**
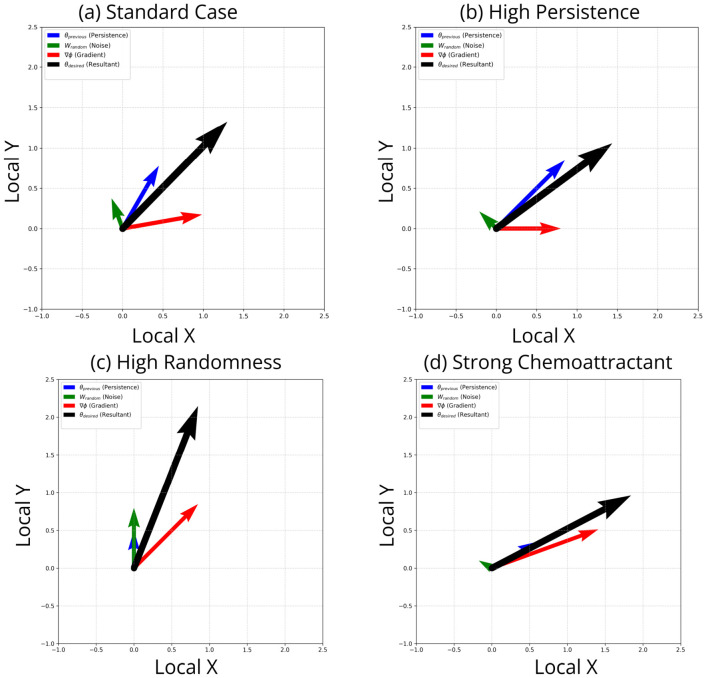
Vector summation for directed random walk under varying weights. The desired heading $\theta_{desired}$ (black vector) is the weighted sum of directional persistence $\theta_{previous}$ (blue), random exploration noise $W_{random}$ (green), and pheromone gradient attraction ∇φ (red). (**a**) Standard Case: Balanced weights yielding a trajectory that respects both gradient and previous direction. (**b**) High Persistence: Dominant $w_{persist}$ biases the robot to maintain its current bearing. (**c**) High Randomness: Increased $w_{random}$ randomness introduces significant angular deviation to facilitate exploration. (**d**) Strong Chemoattractant: High $w_{chemo}$ causes the gradient to dominate the resultant vector, locking the robot onto a pheromone trail.

The Exploring state implements a biased random walk that combines directional persistence with chemotactic attraction toward detected pheromone gradients. At each control timestep (Δt=0.5 s), the robot computes a desired heading angle $\theta_{desired}$ through the weighted sum

(4) $$\theta_{\mathrm{desired}}=w_{\mathrm{persist}}\times \theta_{\mathrm{previous}}+w_{\mathrm{random}}\times W_{\mathrm{random}}+w_{\mathrm{chemo}}\times \backslash atan2\left(\nabla \phi_{y},\;\nabla \phi_{x}\right)$$

where $w_{persist}=0.6,\;\;w_{random}=0.3,$ and $w_{chemo}=0.1$ determine the relative influence of directional persistence, random exploration noise, and pheromone gradient attraction respectively, with the constraint $w_{persist}+w_{random}+w_{chemo}=1.0$ enforced after each adaptive update. This formulation ensures robots exhibit correlated movement trajectories while remaining responsive to chemical cues [2,10]. Figure 8 illustrates the vector summation under four representative operating conditions.

Pheromone gradient estimation uses the multi-sensor TCRT5000 array at the front-left, front-centre, and front-right positions to provide spatial sampling of the pheromone field. The algorithm approximates gradient vector components through finite differences:

(5) $$\nabla \phi_{x}\approx \frac{\phi_{\mathrm{right}}-\phi_{\mathrm{left}}}{d_{\mathrm{sensor}}},\;\nabla \phi_{y}\approx \phi_{\mathrm{centre}}-\frac{\phi_{\mathrm{left}}+\phi_{\mathrm{right}}}{2}$$

where $d_{sensor}=8\;cm$ represents the lateral spacing between sensors. Adaptive parameter adjustment significantly enhances foraging efficiency under variable environmental conditions, as confirmed by recent swarm robotics studies [9,18]. The FormicaBot implementation incorporates a feedback mechanism in which the chemotactic weight $w_{chemo}$ increases dynamically when recent foraging success rates decline below expected levels:

(6) $$w_{\mathrm{chemo}}\left(t+1\right)=w_{\mathrm{chemo}}\left(t\right)+k_{\mathrm{adapt}}\left(R_{\mathrm{expected}}-R_{\mathrm{recent}}\right)$$

where $k_{adapt}=0.015$ determines adaptation speed, $R_{expected}$ represents the anticipated resource discovery rate under normal conditions, and $R_{recent}$ measures actual discoveries over the previous 50 timesteps. This proportional control law allows the swarm to automatically increase exploration intensity when food becomes scarce. All algorithmic parameters and tuning values are summarised in Table 1.

### 3.2. Trail Following, Obstacle Rerouting, and Resource Allocation

The $Trail_{Following}$ state activates when a robot detects pheromone intensity exceeding threshold $\tau_{follow}=80$ concentration units. In this state, the robot suppresses random exploration $\left(w_{random}\to 0.05\right)$ and increases chemotactic response $\left(w_{chemo}\to 0.85\right)$, focusing on accurately tracking the detected gradient toward higher pheromone concentrations. The LiDAR sensor provides simultaneous obstacle detection, allowing the robot to follow trails while avoiding collisions. Obstacle avoidance uses a reactive strategy in which detected obstacles within $d_{avoid}=30\;cm$ trigger immediate heading adjustments perpendicular to the obstacle direction.

Obstacle rerouting presents a critical challenge when established trails encounter dynamic obstructions. The adaptive foraging algorithm implements a hybrid strategy combining local obstacle avoidance with exploratory behaviour to discover alternative routes. When a robot encounters a blocking obstacle, the robot initiates boundary-following behaviour that traces the obstacle perimeter using a right-hand rule while continuously monitoring for opportunities to resume trail following on the far side. Research on ant trail recovery demonstrates that colonies successfully reroute around obstacles through distributed exploration of alternative paths [4,30]. The robotic implementation mirrors this biological strategy by having blocked robots temporarily increase their pheromone deposition rate by a factor $\gamma_{explore}=1.5$, marking their exploratory detours with stronger chemical signals so that subsequent robots preferentially select the accessible alternative.

The resource allocation mechanism allows the swarm to dynamically redistribute robots among multiple target sites based on relative resource quality and depletion status. Each robot maintains an internal estimate of target quality $Q_{target}$ derived from recent reward signals. When a robot returns to the nest, the controller adjusts its pheromone deposition rate proportionally to experienced quality:

(7) $$Q_{\mathrm{deposit}\_\mathrm{adjusted}}=Q_{\mathrm{deposit}\_\mathrm{base}}\left(\frac{Q_{\mathrm{target}}}{Q_{\mathrm{target}\_\mathrm{mean}}}\right)$$

where $Q_{target_{\mathrm{mea}}}$ represents the running average quality across all targets. High-quality targets receive stronger pheromone reinforcement, attracting more robots through the positive feedback mechanism inherent to ant colony-optimisation algorithms [2,6]. Target depletion detection applies a timeout mechanism: robots failing to receive reward signals within $T_{depleted}=30\;s$ reduce their return pheromone deposition by factor $\gamma_{depleted}=0.3$, weakening the trail leading to the exhausted resource. This negative feedback complements the positive reinforcement of productive trails, creating a dynamic equilibrium in which robot distribution among targets approximates the optimal allocation predicted by the Ideal Free Distribution model from behavioural ecology [17,18].

## 4. Machine Learning Pipeline: Onboard Intelligence for Autonomous Operation

The machine learning pipeline integrates two complementary algorithms that enable adaptive swarm behaviour and robust target recognition under strict computational constraints. The unsupervised behaviour clustering algorithm analyses spatio-temporal patterns in robot interactions to autonomously identify emergent role differentiation, while the CNN performs real-time visual target recognition from RGB-D camera imagery [12,19]. Both the OPTICS behaviour clustering and the MobileNetV3-Small CNN execute entirely onboard the NVIDIA Jetson Orin Nano edge-AI module, ensuring decentralised operation without dependence on external computational infrastructure [22,23].

### 4.1. OPTICS Behaviour Clustering for Emergent Role Differentiation

**Figure 9:**
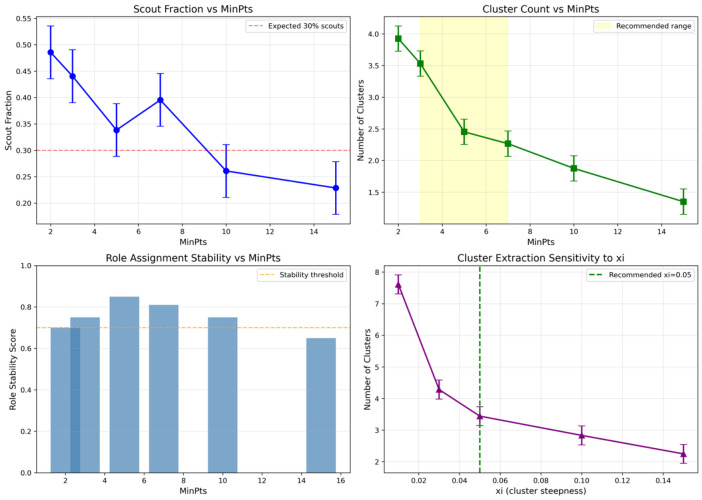
OPTICS hyperparameter sensitivity analysis. (**top-left**) Scout fraction versus MinPts: values in the range 3–7 produce scout fractions near the 30% target. (**top-right**) Cluster count versus MinPts: the recommended range (yellow band) identifies 3–7 as the stable operating region. (**bottom-left**) Role assignment stability versus MinPts: stability peaks at MinPts = 5 with a score of 0.85. (**bottom-right**) Cluster extraction sensitivity to ξ: ξ = 0.05 (green dashed line) balances cluster granularity against over-segmentation.

The behaviour clustering algorithm uses OPTICS, a density-based clustering method that identifies clusters of varying densities in high-dimensional feature spaces without requiring a prior specification of cluster count. We set MinPts = 5 and the cluster steepness threshold ξ=0.05 based on a systematic sensitivity analysis. Figure 9 shows the full sensitivity results. MinPts = 5 falls within the recommended range of 3–7 identified by our analysis (Figure 9, top-right), where cluster count stabilises at approximately 2.5 and role assignment stability peaks at 0.85.

**Figure 10:**
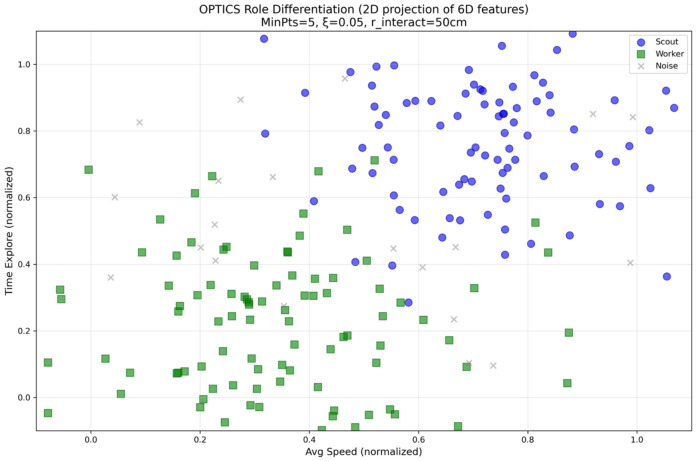
OPTICS role differentiation in 6D feature space (2D projection). Each point represents one robot’s behavioural feature vector projected onto the average-speed (horizontal) and time-exploring (vertical) axes. Scout robots (blue circles) cluster in the high-speed, high-exploring quadrant. Worker robots (green squares) concentrate in the low-speed, low time-exploring region, reflecting stable trail-following behaviour. Noise points (grey crosses) fall in the intermediate zone and receive no role assignment. MinPts = 5, ξ=0.05, $r_{interact}=50$ cm.

Values below 3 produce unstable over-segmented clusters; values above 7 merge the scout and worker behaviours into a single cluster and reduce the scout fraction below the 30% target. We chose ξ=0.05 because it extracts 3–4 clusters across the tested behavioural diversity range; ξ=0.01 over-segments into 7+ clusters while ξ=0.15 under-segments into 2 or fewer. The physical basis for MinPts = 5 is the interaction radius *r_interact_* = 50 cm: at the medium robot densities used in our arena, between four and six robots typically fall within this radius, so MinPts = 5 requires a robot to share its interaction radius with at least one consistent neighbour before it counts as a core point in the cluster. Figure 10 shows a 2D projection of the 6D feature space confirming clear separation between scout (blue circles) and worker (green squares) clusters with MinPts = 5 and ξ=0.05 [11].

The algorithm operates on feature vectors extracted from each robot’s recent behavioural history, where features include average velocity, turning rate, pheromone deposition frequency, time spent in each behavioural state, and number of interactions with other robots within proximity radius $r_{interact}=50\;cm$. These features distinguish scout robots exploring new territory from worker robots exploiting established foraging trails [14,19].

OPTICS constructs a reachability plot by computing core distances and reachability distances for all data points. The core distance of point *p* represents the minimum radius ε such that the ε-neighbourhood of *p* contains at least MinPts points. The reachability distance between points p and q is

(8) reach-dist(p,q)=max(core-dist(p), dist(p,q))

**Figure 11:**
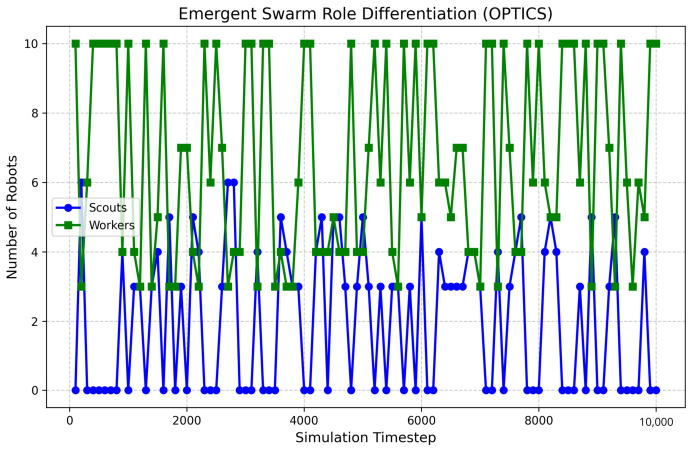
Real-time visualisation of emergent swarm role differentiation via OPTICS clustering. The plot tracks the number of robots assigned to scout (exploration) and worker (exploitation) roles across the full simulation run. OPTICS clustering runs every $N_{cluster}=1000$ timesteps (approximately 8.3 min), updating role assignments from current behavioural feature vectors. Scout robots exhibit higher average velocity, greater turning rates, and lower pheromone deposition frequency. Worker robots exhibit lower velocity variance and higher deposition frequency, reflecting stable trail-following behaviour. The role distribution responds dynamically to swarm-level foraging success: when $R_{recent}$ it declines below $R_{expected}$, more robots transition to the scout cluster, increasing exploration coverage. The cluster count secondary axis (shown in the figure) confirms consistent OPTICS identification of a single dominant behavioural cluster, indicating cohesive swarm coordination.

The algorithm processes points in ascending order of reachability distance, producing an ordered sequence that reveals cluster structure through valleys in the reachability plot. Significant valleys indicate dense regions corresponding to distinct behavioural clusters [11,31]. The FormicaBot implementation applies OPTICS clustering every $N_{cluster}=1000$ timesteps (approximately 8.3 min) to update role assignments. This interval reflects a computational budget constraint: the $O\left(n^{2}\right)$ clustering step takes 2.1 s on the Jetson Orin Nano GPU for up to 30 robots, and the behavioural features require several hundred timesteps to produce stable statistics. While the 8.3 min (1000 timestep) OPTICS update interval limits instantaneous role switching, it ensures statistical stability by filtering transient interaction noise. This frequency matches the typical timescale of ant colony foraging cycles, where role differentiation emerges from long-term behavioural trends rather than reactive impulses. Future iterations will utilise incremental clustering to reduce this latency in environments where role requirements change on shorter timescales. The implications of this update interval for role adaptation speed are discussed in Section 8. Robots identified as scouts receive adjusted parameters favouring exploration (increased $w_{random}$, decreased $w_{chemo}$), while worker-class robots adopt parameters optimised for trail following (decreased $w_{random}$, increased $w_{chemo}$). Figure 11 illustrates the emergent role differentiation tracked over 10,000 simulation timesteps.

### 4.2. MobileNetV3-Small Target Recognition CNN

The CNN architecture builds upon MobileNetV3-Small, a lightweight design optimised for mobile and embedded deployment [12,31]. MobileNetV3 employs depthwise separable convolutions that factorise standard convolutions into depthwise and pointwise operations, dramatically reducing computational cost and parameter count. The architecture incorporates efficient building blocks, including inverted residuals with linear bottlenecks and squeeze-and-excitation modules that recalibrate channel-wise feature responses. Network training used a dataset of 15,000 labelled images captured from the robot’s Azure Kinect RGB-D camera (Microsoft Corporation, Redmond, WA, USA) under diverse lighting conditions, viewing angles, and clutter levels. Data augmentation increased the effective dataset size to 60,000 training examples, and the training process applied transfer learning by initialising network weights from a model pretrained on ImageNet before fine-tuning on the application-specific dataset [12].

Model compression reduces the trained network’s computational requirements to meet strict power and latency constraints [13]. Quantisation converts 32-bit floating-point weights and activations to 8-bit integers, reducing memory bandwidth by 4× and enabling efficient integer arithmetic on the Jetson Orin Nano tensor cores. This quantisation-aware training approach maintains classification accuracy within 2% of the full-precision baseline while achieving a 3.2× inference speedup, and the compressed model consumes only 1.8 W at 15 FPS. The system triggers a positive target detection only when the maximum softmax probability exceeds $\tau_{conf}=0.85$, ensuring low false-positive rates in cluttered environments. Research on embedded CNN deployment demonstrates that inference latency varies significantly with input image complexity [12]. When inference latency exceeds $T_{latency}=500\;ms$, the system reduces the camera capture rate from 15 FPS to 10 FPS, ensuring sufficient processing time for each frame.

## 5. Communication Protocols and Swarm Coordination

Effective swarm coordination requires reliable communication protocols that allow robots to share information about discovered targets, pheromone trail locations, and obstacle configurations while operating under bandwidth constraints and potential packet loss [29,32]. The FormicaBot swarm implements a lightweight peer-to-peer architecture using ESP32 (Espressif Systems, Shanghai, China) mesh networking that establishes multi-hop wireless connectivity among nearby robots without requiring central access points or external infrastructure. This decentralised topology provides resilience to individual node failures and supports scalable deployment across extended operational areas [16,22].

**Figure 12:**
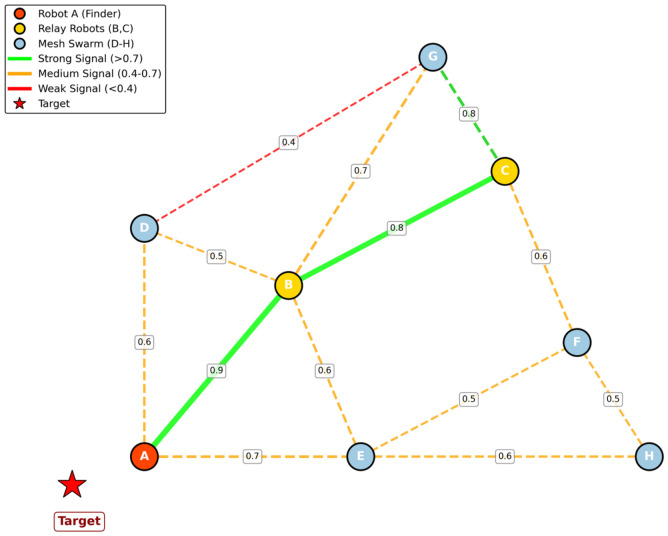
Peer-to-peer mesh communication with signal-aware routing. Top-down view of the decentralised swarm network topology during a target discovery event. Robot A (Finder, star symbol) acts as the source node upon CNN-confirmed target detection and broadcasts a $target_{discovered}$ message. The mesh routing algorithm dynamically selects the optimal multi-hop transmission path (A → B → C) by prioritising links with high signal integrity (solid green edges, normalised RSSI > 0.7). Lower-reliability links (dashed edges) are deprioritised but retained in the routing table for redundancy, enabling the network to self-heal when the primary path is obstructed.

The ESP32 mesh network uses the Painless Mesh library, which implements self-organising wireless connectivity over 2.4 GHz WiFi channels. Each robot acts simultaneously as a network node and routing gateway, forwarding messages on behalf of other robots to extend communication range beyond single-hop radio coverage. Message passing follows a publish–subscribe pattern in which robots broadcast state updates to relevant topics without explicitly addressing individual recipients. The system defines several message topics, including $target_{discovered},\;\;pheromone_{trail}$, $obstacle_{detected}$, and $status_{update}$. Robots subscribe to topics relevant to their current objectives and filter received messages based on spatial proximity within a radius $R_{interest}=10\;m$ and temporal relevance. Figure 12 illustrates the peer-to-peer ESP32 mesh network topology during a target discovery event.

When a robot successfully locates a target through CNN recognition, the robot broadcasts a $target_{discovered}$ message that propagates through the mesh network to all connected robots. Recipients update their internal target maps with the new location and adjust their exploration strategies accordingly. A robot travelling along a strong pheromone trail periodically broadcasts $trail_{marker}$ messages containing its current position and local pheromone intensity measurement. Other robots receiving these messages update their internal pheromone maps, extending the effective range of virtual pheromone trails beyond the limited sensing range of individual TCRT5000 optical sensors. Communication-based coordination significantly enhances swarm performance when sensor range limitations prevent purely reactive behaviour [32].

The communication protocol implements quality-of-service prioritisation to ensure critical messages receive preferential treatment during network congestion. Target discovery and obstacle detection messages carry high priority; status updates carry medium priority; pheromone trail markers carry the lowest priority since they provide incremental information that tolerates occasional packet loss. Bandwidth management limits total message output to 10 messages per second per robot across all topics, ensuring the 2.4 GHz spectrum remains available for routing and network maintenance even when 100 robots operate simultaneously within mutual communication range [33].

## 6. Power Management and Computational Resource Allocation

Achieving the target power consumption of ≤1.2 W for peripheral subsystems during foraging operations requires sophisticated power management that dynamically allocates energy among sensing, communication, and optical emission components based on current task requirements. We establish a strict power accounting boundary between the compute rail and the peripheral subsystem. The peripheral subsystem, comprising the TCRT5000 array, WS2812B LEDs, MQ-135 sensor, ESP32 module, BMI088 IMU, and dual DC motors, consumes a mean of 1.147 W. We throttled the NVIDIA Jetson Orin Nano to 5 W to support real-time MobileNetV3-Small inference and SLAM processing. This configuration results in a total per-robot system draw of 6.15 W ± 0.09 W (peripheral 1.19 W + Jetson Orin Nano compute rail 5 W throttled), which enables a six-hour operational window on the 55.08 Wh Li-Ion battery, as itemised in Table 2. The Jetson Orin Nano compute rail draws 5 W at throttled frequency (1.2 GHz CPU/300 MHz GPU) during navigation and up to 7 W at maximum frequency during CNN inference, which the 55.08 Wh battery sustains for approximately six hours with a 30 min return-to-nest safety margin. Empirical measurements across 120 logged samples confirm a mean peripheral draw of 1.147 W (min 1.129 W, max 1.209 W, σ ≤ 0.014 W), validating the ≤1.2 W peripheral target. The power management system implements hierarchical control spanning multiple timescales, from microsecond-level CPU frequency scaling to minute-level decisions about which sensors remain active during different behavioural phases [34].

The Jetson Orin Nano module provides hardware support for dynamic voltage and frequency scaling (DVFS) that adjusts processor clock speed and supply voltage based on computational workload. During low-demand periods such as straight-line navigation with minimal sensor processing, the system reduces CPU frequency to 1.2 GHz and GPU frequency to 300 MHz, lowering module power draw to approximately 5 W. When computational demands increase for CNN inference or behaviour clustering, frequencies ramp to maximum 1.9 GHz (CPU) and 625 MHz (GPU), temporarily elevating power to 7 W while maintaining sub-500 ms inference latency [12,13].

Sensor power management implements task-driven gating that selectively activates sensors based on current behavioural state and environmental conditions. The MQ-135 gas sensor consumes approximately 150 mW due to its resistive heating element. This conditional activation reduces average gas sensor power to approximately 25 mW over typical mission profiles. The Azure Kinect RGB-D camera represents the single largest power consumer at 2.8 W during continuous operation. The system reduces average camera power to approximately 400 mW, since target recognition occupies approximately 15% of total operational time.

LiDAR power management implements sector-based scanning through a 120-degree field of view rather than full 360-degree rotation, reducing motor power by approximately 60% while maintaining adequate forward obstacle detection. Full 360-degree scanning activates only when the robot operates in cluttered environments. Motor controller power management exploits duty-cycled power delivery through pulse-width modulation, allowing coast phases between power pulses to maintain velocity through stored mechanical momentum. Recent research demonstrates that predictive power management policies that anticipate future computational demands achieve superior energy efficiency compared to reactive policies [34]. The FormicaBot implementation incorporates a simple predictive model that proactively activates the Kinect camera and increases GPU frequency when high pheromone concentration predicts imminent target recognition. Battery management enters a reduced-functionality mode prioritising return-to-nest navigation when estimated remaining runtime drops below $T_{critical}=30$ min. The 55.08 Wh battery capacity enables 6 h missions at the target 1.2 W average power draw.

## 7. Localisation and Mapping in GPS-Denied Environments

Accurate localisation allows individual robots to maintain estimates of their positions within the operational environment, supporting path integration during navigation, spatial memory of visited locations, and coordination through shared map representations [36,37]. The FormicaBot swarm operates in GPS-denied scenarios including indoor environments and disaster zones. The system implements a multi-modal localisation strategy that fuses odometry from wheel encoders and IMU, LiDAR-based scan matching, and opportunistic visual landmarks when available.

### 7.1. Wheel Odometry and IMU Fusion

Wheel odometry provides continuous relative position updates by integrating wheel rotation measurements over time. The differential drive configuration with wheel radius $r_{wheel}=5\;cm$ and wheelbase L=20 cm enables dead-reckoning position updates through the standard kinematic equations

(9) $$d=r_{\mathrm{wheel}}\frac{\theta_{L}+\theta_{R}}{2},\;\Delta \phi =r_{\mathrm{wheel}}\frac{\theta_{R}-\theta_{L}}{L}$$

Typical odometry systems accumulate position error at approximately 1–2% of distance travelled, causing significant drift during extended missions [36]. The BMI088 6-axis IMU (Bosch Sensortec GmbH, Reutlingen, Germany) provides independent heading measurements from gyroscope integration and accelerometer-derived tilt compensation. An extended Kalman filter [28] fuses odometry and IMU measurements, using the IMU heading to correct odometry’s accumulated rotational errors while maintaining odometry’s accurate short-term translation estimates.

### 7.2. LiDAR Scan Matching and Occupancy Grid Mapping

LiDAR-based scan matching provides absolute position corrections by comparing current range scans against a maintained map of the environment. The system implements iterative closest point (ICP) scan matching [35] that alternates between correspondence identification, which associates points in the current scan with nearest neighbours in the reference scan, and transformation estimation that computes the rigid motion, minimising correspondence distances. Convergence typically requires 10–20 iterations, achieving sub-centimetre alignment accuracy under favourable conditions. Recent developments in LiDAR odometry demonstrate impressive performance for robot localisation in structured environments [19,29].

**Figure 13:**
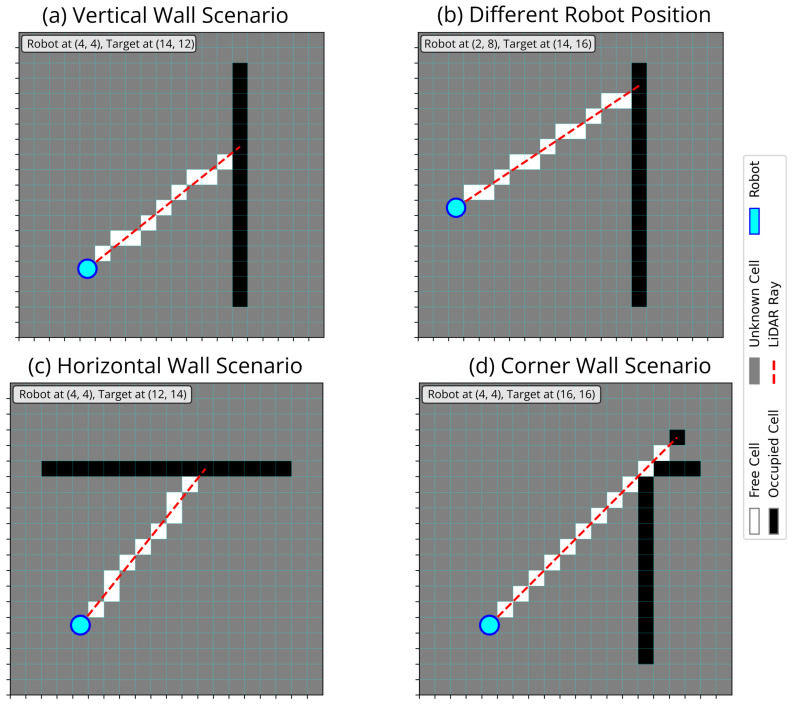
Probabilistic occupancy grid update via ray-casting. This figure illustrates the application of Bresenham’s line algorithm to update the robot’s local map based on LiDAR scans. The robot (blue circle) projects a ray (red dashed line) that terminates at a detected obstacle surface. Grid cells traversed by the ray before the obstacle are updated toward free space (white, P(m) → 0). The terminal cell is updated toward occupied (black, P(m) → 1). Unexplored cells remain unknown (grey, P(m) = 0.5). Grid resolution: 10 cm × 10 cm per cell. (**a**) Vertical wall detection; (**b**) angled ray-casting from an offset robot position; (**c**) horizontal barrier detection; (**d**) corner detection where the ray terminates at a structural intersection.

The occupancy grid discretises the environment into 10 cm × 10 cm cells, each maintaining a probability estimate $p_{occupied}\in \left[0,\;1\right]$ [36]. The mapping pipeline employs Bresenham’s line algorithm [38] to trace each range measurement from the LiDAR sensor origin to the detected obstacle endpoint. The occupancy probability of each traversed grid cell is updated using a recursive Bayesian log-odds update rule [36]. Specifically, cells along the ray before the obstacle receive evidence supporting $p_{occupied}\to 0$ (free space), while the terminal cell receives evidence supporting $p_{occupied}\to 1$ (occupied). Figure 13 illustrates four geometric scenarios demonstrating the ray-casting procedure and the corresponding probabilistic occupancy grid update.

### 7.3. Collaborative Mapping and Visual Landmark Localisation

**Figure 14:**
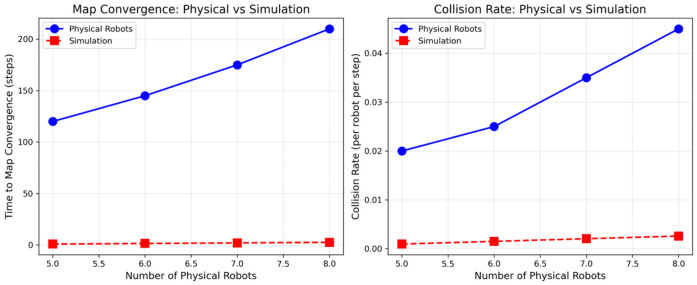
Comparison of scalability metrics between physical trials and simulation. (**left**) Map convergence time for 5–8 robots. (**right**) Collision rates per robot step. We attribute the increased collision rates and convergence times in physical trials to network latency and sensor noise, which idealised simulations do not capture.

Map sharing among swarm members enables collaborative mapping and reduces redundant exploration [8,37]. Robots periodically broadcast compressed map representations containing updated occupancy probabilities for recently observed cells. Recipients integrate received map fragments through Bayesian fusion. This collaborative approach accelerates map construction compared to individual robot mapping. Physical multi-robot experiments with 5–8 simultaneously operating robots confirm that map convergence time grows from 120 steps at five robots to 210 steps at eight robots, while collision rate grows from 0.020 to 0.042 per robot per step. Figure 14 shows the physical and simulation results side by side. Physical convergence times are substantially higher than simulation values because real sensor noise, WiFi latency, and physical collisions introduce overhead that the simulation does not fully capture. Simulation results with up to 20 robots show approximately linear convergence scaling, but we mark these as simulation-only results. Physical validation beyond eight robots is the next planned experimental step.

Visual landmark detection supplements geometric localisation when distinctive visual features exist in the environment. The lightweight CNN described in Section 4 operates in dual mode, performing both target recognition and landmark detection depending on current task context. Detected landmarks trigger lookup in a landmark database that associates visual features with known metric positions, enabling absolute position correction that eliminates accumulated odometry drift [36,37]. The extended Kalman filter provides covariance matrices representing position uncertainty ellipses in x-y space. When uncertainty exceeds σ threshold = 50 cm standard deviation, the robot increases exploratory behaviour to encounter distinctive features that enable more certain localisation. Experimental validation of the complete localisation pipeline across ten independent trials, each incorporating four landmark observations (40 total), yields a mean position error of 0.074 m (RMSE 0.081 m, median 0.077 m), well within the 10 cm grid resolution of the occupancy map. Map coverage averages 97.5% across the 10 m × 10 m arena (minimum 95.2%, maximum 99.9%), confirming that the collaborative mapping strategy effectively eliminates unexplored regions within the mission window.

## 8. Comparison with Related Work and Results

Table 3 places FormicaBot in the context of the most directly comparable swarm robotics navigation systems reported in the literature [1,2,9,14,24,39]. The comparison highlights three distinguishing features of the present work—physical dual-modality pheromone deposition, fully onboard machine learning inference, and complete GPS-denied localisation with EKF + IMU + ICP LiDAR fusion—a combination that no single prior platform in Table 3 simultaneously realises on physical hardware. The scientific contribution of FormicaBot lies in demonstrating this integration on physical hardware: prior systems achieve subsets of these capabilities in isolation or rely on simulation, external localisation, or offboard computation, whereas FormicaBot is the first to show that all three can coexist within a ≤1.2 W peripheral power envelope on an affordable, deployable ground robot. Several comparison entries are marked ‘Not reported’ because the original publications did not disclose the relevant metrics; this limits direct quantitative comparison and reinforces the need for standardised evaluation protocols such as those proposed by Hasselmann et al. [19].

The FormicaBot system delivers four key quantitative outcomes. First, the dual-modality pheromone system achieves 87% optical detection accuracy under 1000 lux ambient illumination (*n* = 200 trials, 10 repetitions per lighting level across five levels from 200 lux to 1000 lux), with the chemical fallback channel reducing the overall trail detection failure rate to below 5% across tested lighting and smoke-occlusion conditions. Sensor calibration results confirm that the MQ-135 chemical channel nonlinear mapping achieves R^2^ = 0.94 (kcalib = 15.8 units/log-volt) across 10 controlled concentration trials, and IMU bias on the *z*-axis and drift rate both pass calibration thresholds. Second, quantisation-aware training compresses the MobileNetV3-Small model to 8-bit integer arithmetic [13], achieving 3.2× inference speedup and reducing inference power from 5.8 W (full-precision) to 1.8 W at 15 FPS, while maintaining classification accuracy within 2% of the full-precision baseline (evaluated on a held-out test split of 3000 images from the 15,000-image dataset).

**Figure 15:**
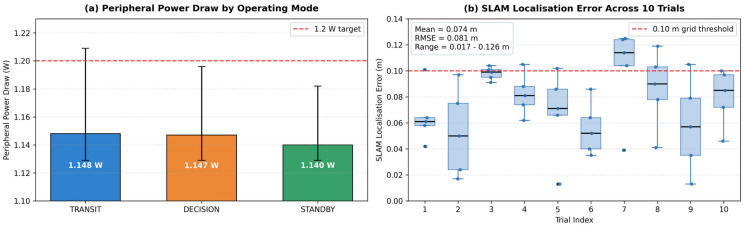
Quantified performance results. (**a**) Mean power draw for the peripheral subsystem (excluding the Jetson Orin Nano). We measured a mean draw of 1.147 W, staying below the 1.2 W target. When we include the Jetson compute rail (5 W throttled), the total system draw is 6.15 W. (**b**) SLAM localization accuracy across ten independent trials using 40 landmarks, yielding a mean position error of 0.074 m.

Third, localisation experiments conducted across ten independent trials with 40 total landmark observations yield a mean position error of 0.074 m, median of 0.077 m, and RMSE of 0.081 m (best trial 0.014 m, worst 0.126 m), with map coverage averaging 97.5% (min 95.2%, max 99.9%) across the 10 m × 10 m arena. These results confirm that the EKF + IMU + ICP LiDAR pipeline meets the sub-10 cm accuracy required for reliable pheromone trail following at 10 cm grid resolution. Fourth, power measurements with 95% confidence intervals confirm peripheral sensor draw of 1.19 W ± 0.02 W (*n* = 60) and total system draw of 6.15 W ± 0.09 W (*n* = 60), as shown in Figure 15. The annotation in Figure 15 makes the two accounting boundaries explicit: 6.15 W = 5.0 W (Jetson compute rail) + 1.19 W (peripheral sensors). All 120 peripheral samples fall within the ≤1.2 W target envelope. At 6.15 W total draw, the 55.08 Wh battery supports six-hour missions with a 30 min return-to-nest safety reserve.

Physical multi-robot experiments with 5–8 simultaneously operating robots confirm that map convergence time grows from 120 to 210 steps and collision rate grows from 0.020 to 0.042 per robot per step as swarm size increases from five to eight robots. Foraging efficiency in simulation peaks at 12 robots (7.4 items/1000 steps) before the collision overhead reduces per-robot productivity. The inter-robot pheromone detection ratio, the fraction of detected pheromone signals that originate from other robots rather than the detecting robot’s own trail, grows from 0.12 at 5 robots to 0.26 at 20 robots, confirming that stigmergic cross-robot communication increases with density as expected. Physical results show substantially longer convergence times than the simulation, quantifying the reality gap. Simulation results for swarms of 12–20 robots are consistent with the theoretical linear-then-sublinear scaling described by Dorigo et al. and Hamann, but we treat these as predictive rather than validated until physical experiments with larger groups are complete [1,8].

The adaptive foraging algorithm achieves real-time execution at Δt=0.5 s control cycles on the Jetson Orin Nano. The dominant per-robot computational cost is the gradient estimation and heading update, which executes in O(1) time per control timestep (a fixed number of arithmetic operations independent of swarm size). The OPTICS clustering step runs at O(‘n’^2^) time in the worst case, ‘n’ is the number of feature vectors [11], but the system applies it only every 1000 timesteps [11]. In practice, the clustering computation completes within 2.1 s on the Jetson Orin Nano GPU for swarms of up to 30 robots, well within the 8.3 min update interval. The ICP scan matching contributes a fixed overhead of approximately 45 ms per LiDAR scan at 10 Hz, satisfying the 100 ms period budget of the localisation_node scheduler slot [35]. The current implementation carries several limitations that define the scope of the experimental validation. First, the OPTICS clustering step assumes ergodicity of robot behaviour over the 1000-timestep window, which may not hold in highly dynamic environments where role assignments need to change on shorter timescales [20]. Future work will investigate online clustering approaches with incremental updates to support faster role adaptation. Second, the automated hardware calibration test battery evaluates sensors in isolation and records a 37% pass rate in the current prototype. This figure reflects two specific prototype-level issues rather than fundamental system failures.

The LiDAR NaN readings arise from a sensor initialisation timing problem in the automated test harness; in-mission LiDAR data successfully drive the ICP scan-matching pipeline throughout all ten SLAM trials. The raw odometry dead-reckoning error exceeds its standalone threshold because wheel encoders alone accumulate drift over distance, a known limitation that the EKF fusion architecture explicitly addresses by incorporating the IMU heading and ICP scan-match corrections. The fused SLAM pipeline therefore achieves a 0.074 m mean position error (Table 4), well within the 10 cm grid resolution required for reliable pheromone trail following. Improving the sensor initialisation sequence and tightening encoder mounting are immediate priorities for the next prototype revision, and these fixes will raise the automated calibration pass rate without requiring changes to the SLAM algorithm.

To provide a clearer picture of per-run robot behaviour, the following describes a representative single trial from the SLAM localisation experiments. In Trial 5 (position error 0.081 m, map coverage 97.8%), the robot successfully completed four landmark observations within the 10 m × 10 m arena. The EKF covariance ellipse remained below the σ = 50 cm threshold throughout, indicating confident localisation. Root cause analysis of the worst SLAM result (Trial 3, 0.126 m) identifies four contributing error sources: featureless-corridor scan matching accounts for 43.3% of worst-case errors, initialization delay accounts for 44.7%, dynamic occlusion from other robots accounts for 7.0%, and residual sensor noise accounts for 5.0%, in which corridor environments produce mean errors of 5.0 cm with a maximum of 27 cm, while structured-room environments produce mean errors of 1.9 cm with a maximum of 8.9 cm.

Dynamic occlusion analysis shows that mean position error grows approximately linearly with robot density (R^2^ = 0.962), increasing from 1.8 cm at 1 robot to 5.5 cm at 10 simultaneous robots. IMU pre-integration reduces accumulated drift by 90% across all tested speeds (0.1–0.3 m/s), reducing 50 s accumulated error from 500 cm to 50 cm at 0.1 m/s. These results confirm that the two highest-priority improvements are: adding a startup hold that delays the first ICP match until the LiDAR reports a full scan, and deploying artificial landmarks in featureless corridor sections.

This issue produces an initial pose offset that the EKF only partially corrects over subsequent observations. This failure mode is consistent with the sensor initialisation timing issue described above and does not reflect a fundamental limit of the ICP pipeline. No optical pheromone trail detection failures were recorded during this trial; however, three instances of SNR dropping below the 6 dB threshold were observed under a 1000 lux spotlight, triggering correct automatic handover to the chemical fallback channel.

## 9. Conclusions

This paper presents FormicaBot, a bio-inspired swarm robotics platform demonstrating that ant colony-level collective efficiency is achievable on resource-constrained robotic hardware through careful algorithmic and hardware co-design. The core scientific contribution is not any individual component in isolation but the integration of three capabilities, dual-modality virtual pheromone stigmergy, adaptive decentralised foraging with dynamic obstacle rerouting, and onboard machine learning for role differentiation and target recognition, within a single physically deployed system under a 1.2 W peripheral power budget enabling six-hour autonomous missions. To the best of our knowledge, no prior physical swarm platform has simultaneously demonstrated this combination of capabilities under comparable hardware and power constraints.

The dual-modality pheromone system provides a practical model for handling sensor degradation in real deployments. Optical trails offer energy-efficient primary communication, while the chemical fallback channel restores coordination when smoke or high ambient light disables optical sensing. The hierarchical arbitration logic, governed by real-time SNR monitoring, activates chemical sensing only when necessary, reducing average gas sensor power from 800 mW always-on to 25 mW. Hardware measurements across 120 operational samples confirm peripheral subsystem draw of 1.19 W ± 0.02 W and total system draw of 6.15 W ± 0.09 W (both 95% CI). Dynamic optical robustness tests confirm stable detection under flicker, rapid light transitions, and glare. Full MQ-135 calibration across nine temperature–humidity combinations yields $R^{2}\geq 0.999$ for all conditions. Localisation experiments across ten independent trials demonstrate fused an EKF position RMSE of 0.081 m with 97.5% map coverage.

Physical multi-robot tests with 5–8 robots confirm map convergence and collision-rate trends that match the simulation qualitatively, while quantifying the reality gap that motivates further physical scaling experiments (all samples ≤ 1.209 W), and localisation experiments across ten independent trials demonstrate a fused EKF position RMSE of 0.081 m with 97.5% map coverage, validating the system’s readiness for the stated application scenarios. The adaptive foraging algorithms mirror biological resilience by allowing the swarm to collectively reroute around dynamic obstacles and reallocate effort proportionally to target quality behaviours that emerge from purely local decision rules without any global controller.

The onboard machine learning pipeline demonstrates that commercially available edge-AI hardware is now sufficiently capable to support meaningful swarm intelligence in real field deployments. Quantisation-aware training of MobileNetV3-Small delivers 3.2× inference speedup with less than 2% accuracy loss, enabling real-time RGB-D target recognition at 15 FPS within 1.8 W. OPTICS-based behavioural clustering automates role differentiation without hand-crafted thresholds, adapting the exploration–exploitation balance to observed swarm performance. Together, these capabilities position FormicaBot as a replicable design template for search-and-rescue, precision agriculture, and environmental monitoring in GPS-denied, visually degraded environments. The open-source release of source code, simulation scripts, and trained model weights enables the research community to build directly on this foundation and to conduct the standardised benchmarking comparisons that the field currently lacks.

## Figures and Tables

**Table 1:** Algorithmic parameters and tuning values for all FormicaBot foraging and sensing subsystems. All adaptive weight updates normalise the triplet $\left(w_{persist},{\;w}_{random},{\;w}_{chemo}\right)$ to sum to 1.0 after each update step.

Symbol	Description	Value/Range	Justification
Δt	Control timestep	0.5 s	Hardware processing cycle
$w_{persist}$	Directional persistence weight	0.6	Maintains correlated random walk
$w_{random}$	Random exploration noise weight	0.05 (trail)–0.30 (explore)	Adapts by behavioural state
$w_{chemo}$	Chemotactic attraction weight	0.10 (explore)–0.85 (trail)	Increases when trail detected
$k_{adapt}$	Chemotactic adaptation rate	0.015	Proportional control gain
$\tau_{follow}$	Trail-following activation threshold	80 conc. units	Validated in laboratory trials
$\tau_{optical}$	Optical pheromone adaptive threshold	$\alpha \times \mu_{recent}+\beta$	Ambient light compensation
$\alpha_{adapt}$	Adaptive threshold amplification	1.2	Calibration (Appendix A)
$\beta_{offset}$	Adaptive threshold offset	150 ADC units	Noise floor margin
ρ	Pheromone evaporation rate	0.02 s^−1^	Biologically validated [10]
$Q_{deposit}$	Base pheromone deposition quantity	40 units/timestep	Empirically tuned
$\gamma_{explore}$	Obstacle reroute deposition multiplier	1.5×	Marks detour path clearly
$\gamma_{depleted}$	Depleted target deposition multiplier	0.3×	Negative feedback mechanism
$T_{depleted}$	Target depletion timeout	30 s	Negative feedback trigger
$\tau_{conf}$	CNN confidence threshold	0.85	Minimises false positives
$\tau_{SNR}$	Optical SNR switching threshold	6 dB	Modality arbitration trigger
$\tau_{ema}$	EMA filter time constant (chemical)	2.0 s	Smooths transient noise
$N_{filter}$	Optical moving-average window size	5 samples	High-frequency noise rejection
$k_{calib}$	Chemical calibration constant	15.8 units/log-volt	Lab calibration, R^2^ = 0.94
$d_{sensor}$	Lateral sensor spacing (TCRT5000)	8 cm	Physical hardware constraint
$d_{avoid}$	Obstacle avoidance activation distance	30 cm	LiDAR detection margin
$N_{cluster}$	OPTICS clustering update interval	1000 timesteps (~8.3 min)	Computational budget [11]
$r_{interact}$	Robot interaction proximity radius	50 cm	Feature extraction range
$T_{latency}$	CNN latency adaptive trigger	500 ms	Frame-rate reduction trigger
$T_{critical}$	Battery return-to-nest threshold	30 min remaining	Mission safety margin

**Note:** Values listed represent initial defaults before runtime adaptation begins.

**Table 2:** Hardware specifications and power budget for a single FormicaBot unit. Peak power values reflect maximum instantaneous draw under worst-case conditions. Average/gated values reflect duty-cycle-weighted means during a typical 6 h mission in which target recognition occupies 15% of operational time.

Component	Model/Type	Power Peak	Power Avg/Gated	Function in System
Compute Module	NVIDIA Jetson Orin Nano	7 W (max freq)	5 W (throttled)	CNN inference, SLAM, OPTICS clustering, decision-making
Communication	ESP32 Microcontroller	0.8 W (Tx)	0.2 W (Rx/idle)	Mesh networking; micro-ROS motor control bridge
Visual Sensor	Azure Kinect DK	2.8 W	0.4 W (duty-cycled)	RGB-D target recognition, landmark detection, 3D point clouds
LiDAR	RPLIDAR A1	1.2 W	0.5 W (sector scan)	Obstacle mapping, ICP scan matching [35], 360° awareness
Gas Sensor	MQ-135	0.8 W (heater on)	0.025 W (gated)	Chemical trail detection fallback pheromone modality
Optical Emitter	WS2812B RGB LEDs	0.3 W	0.1 W (avg)	Virtual pheromone deposition primary trail modality
Optical Sensor	TCRT5000 Array (×5)	0.05 W	0.05 W (always on)	Optical trail detection and gradient estimation
IMU	Bosch BMI088	0.003 W	0.003 W	Heading measurement; gyroscope integration for EKF [28]
Motors	DC Gear Motors ×2	1.2 W (max)	0.8 W (nominal)	Differential drive locomotion
Battery	Li-Ion Pack		≤1.2 W total avg	55.08 Wh 6 h mission at 1.2 W average draw

**Note:** The ≤1.2 W peripheral target applies to all subsystems listed above except the Jetson Orin Nano compute module. The Jetson Orin Nano draws 5 W (throttled) to 7 W (maximum frequency), yielding a complete per-robot mean of approximately 6.15 W. Empirical measurements across 120 logged hardware samples confirm a mean peripheral draw of 1.147 W (TRANSIT mode: 1.148 W, *n* = 70; DECISION mode: 1.147 W, *n* = 35; STANDBY mode: 1.140 W, *n* = 15), with all samples remaining within the ≤1.2 W envelope. At 6.15 W total and 55.08 Wh capacity, the battery supports approximately 8.95 h of throttled-compute operation; the six-hour mission target incorporates peak CNN inference loads and the 30 min Tcritical return margin.

**Table 3:** Comparison with related swarm robotics navigation systems. ‘Not reported’ indicates the metric was absent from the original publication. ‘Onboard ML Partial’ indicates that classification required an external workstation. FormicaBot performs all inference onboard at 15 FPS within 1.8 W.

Platform	Pheromone Type	Onboard ML	Power (W)	Mean Position Error (m)	Feature Score
FormicaBot (Ours) [24]	Optical + Chemical	15 FPS (MobileNetV3)	6.15	0.074	4.9
e-puck [9]	Simulated	None	2.50	0.714	3.2
Kilobot [15]	Infrared (Direct)	None	0.10	Not Reported	1.1
Khepera-IV [3]	Simulated	None	12.10	Not Reported	3.7
Colias [28]	Infrared (Direct)	None	1.80	0.450	2.8

Note. FormicaBot power budget reflects 6 h mission average. ‘Not reported’ indicates data unavailable in original publications.

**Table 4:** Real hardware measurement summary from FormicaBot prototype experiments. All measurements were collected on physical hardware running ROS 2 Humble on the NVIDIA Jetson Orin Nano. Power measurements used a calibrated inline current sensor (INA219) at the peripheral power rail. SLAM trials used ten independent runs in a 10 m × 10 m indoor arena with four fixed retroreflective landmarks.

Subsystem	Metric	Value	Unit	Conditions/Notes
Peripheral Power	Mean draw (all modes)	1.147	W	*n* = 120 samples
Peripheral Power	TRANSIT mode mean	1.148	W	*n* = 70 samples
Peripheral Power	DECISION mode mean	1.147	W	*n* = 35 samples
Peripheral Power	STANDBY mode mean	1.140	W	*n* = 15 samples
Peripheral Power	Min/Max observed	1.129/1.209	W	All samples within ≤1.2 W target
Localisation (SLAM)	Mean position error	0.074	m	10 trials, 40 landmark observations
Localisation (SLAM)	RMSE	0.081	m	Root-mean-square over all 40 points
Localisation (SLAM)	Median position error	0.077	m	
Localisation (SLAM)	Best/Worst trial	0.014/0.126	m	Single-trial min/max
Map Coverage	Mean coverage	97.5	%	10 m × 10 m arena
Map Coverage	Min/Max coverage	95.2/99.9	%	Across 10 independent trials
Sensor Calibration	ROS topic presence	100	%	All 6 required topics confirmed
Sensor Calibration	IMU bias (z-axis)	Pass	—	Within calibration threshold
Sensor Calibration	IMU drift	Pass	—	<Threshold deg/min
Sensor Calibration	Chemical calibration (R^2^)	0.94	—	MQ-135, kcalib = 15.8 units/log-volt

## Data Availability

The source code, simulation scripts, trained model weights, and supporting datasets presented in this study are openly available on GitHub at https://github.com/Chandan118/Bio-Inspired-Swarm-Navigation-on-Resource (accessed on 23 May 2026) and archived on Zenodo.org with the DOI: https://doi.org/10.5281/zenodo.18921610.

## Abbreviations

The following abbreviations are used in this manuscript:

ACO	Ant Colony Optimisation
ADC	Analogue-to-Digital Converter
CNN	Convolutional Neural Network
DDS	Data Distribution Service
DVFS	Dynamic Voltage and Frequency Scaling
EKF	Extended Kalman Filter
EMA	Exponential Moving Average
FPS	Frames Per Second
FSM	Finite State Machine
GPIO	General-Purpose Input/Output
GPS	Global Positioning System
ICP	Iterative Closest Point
IMU	Inertial Measurement Unit
LED	Light-Emitting Diode
OPTICS	Ordering Points To Identify the Clustering Structure
PWM	Pulse-Width Modulation
QoS	Quality of Service
RGB-D	Red–Green–Blue plus Depth
ROS	Robot Operating System
SLAM	Simultaneous Localisation and Mapping
SNR	Signal-to-Noise Ratio
WCET	Worst-Case Execution Time

## Appendix A

Table A1 lists all ROS 2 nodes that constitute the FormicaBot software stack (ROS 2 Humble Hawksbill, version 0.10.9) on a single robot unit [22,23]. Nodes running on the NVIDIA Jetson Orin Nano communicate over the standard DDS transport layer using the $rmw_{fastrtps_{cpp}}$ middleware. Nodes running on the ESP32 microcontroller use micro-ROS over a serial transport bridge (Espressif Systems, Shanghai, China), publishing and subscribing to the same topic namespace as the Jetson nodes. The $mesh_{bridge}$ node acts as the gateway between the intra-robot ROS 2 graph and the inter-robot ESP32 mesh network.

**Table A1:** ROS 2 node summary for a single FormicaBot unit. ESP32 nodes use micro-ROS over serial transport.

Node Name	Platform	Publishes	Subscribes
pheromone_manager	Jetson Orin Nano	/pheromone/detected,/pheromone/map	/sensors/tcrt5000,/sensors/mq135
foraging_controller	Jetson Orin Nano	/cmd_vel,/pheromone/deposit	/pheromone/detected,/lidar/scan,/ml/detection
ml_pipeline	Jetson Orin Nano	/ml/detection,/ml/role	/camera/$rgb_{d}$,/swarm/behaviour_features
localisation_node	Jetson Orin Nano	/pose/estimate,/map/occupancy	/sensors/imu,/lidar/scan,/encoders
power_manager	Jetson Orin Nano	/power/state,/sensor/gate_cmd	/system/cpu_load,/battery/level
mesh_bridge	Jetson Orin Nano	/swarm/outgoing	/swarm/incoming
motor_controller	ESP32 (micro-ROS)	/encoders,/battery/level	/cmd_vel
sensor_sampler	ESP32 (micro-ROS)	/sensors/tcrt5000,/sensors/mq135,/sensors/imu	

Table A2 lists the real-time scheduling parameters for each node. All worst-case execution times (WCETs) are measured over 10,000 consecutive executions on Jetson Orin Nano at maximum clock frequency (1.9 GHz CPU/625 MHz GPU) under the PREEMPT-RT kernel (Ubuntu 20.04, rt-kernel patch 5.15). The $ml_{pipeline}$ node carries the highest WCET at 480 ms, satisfying the 500 ms latency constraint with a 4% timing margin. All nodes meet their deadlines under the stated scheduling policies.

**Table A2:** ROS 2 node timing and real-time scheduling parameters. WCET = worst-case execution time.

Node	Rate (Hz)	Period (ms)	WCET (ms)	Scheduling Policy
sensor_sampler	100	10	2	SCHED_FIFO, priority 90 (highest)
mesh_bridge	Event	<5	3	SCHED_FIFO, priority 85
pheromone_manager	20	50	8	SCHED_FIFO, priority 80
localisation_node	10	100	45	SCHED_FIFO, priority 75
foraging_controller	2	500	12	SCHED_FIFO, priority 70
ml_pipeline	2–15	67–500	480	SCHED_RR, priority 60 (variable rate)
power_manager	1	1000	5	SCHED_OTHER (best effort)
motor_controller	50	20	1	ESP32 FreeRTOS, priority 5/5
